# Atomic Force Microscopy Nanoindentation Method on Collagen Fibrils

**DOI:** 10.3390/ma15072477

**Published:** 2022-03-27

**Authors:** Stylianos Vasileios Kontomaris, Andreas Stylianou, Anna Malamou

**Affiliations:** 1 Faculty of Engineering and Architecture, Metropolitan College, 15125 Athens, Greece; 2 BioNanoTec LTD, 2043 Nicosia, Cyprus; 3 Cancer Mechanobiology and Applied Biophysics Group, Basic and Translational Cancer Research Center, School of Sciences, European University Cyprus, 2404 Nicosia, Cyprus; 4 Radar Systems and Remote Sensing Lab, School of Electrical and Computer Engineering, National Technical University of Athens, 15780 Athens, Greece; annamalamou@yahoo.gr

**Keywords:** Atomic Force Microscopy (AFM), nanomechanical properties, Young’s modulus, Hertz model, Oliver–Pharr analysis, linear elasticity, mechanical heterogeneous samples, biological samples, nanoscale

## Abstract

Atomic Force Microscopy nanoindentation method is a powerful technique that can be used for the nano-mechanical characterization of bio-samples. Significant scientific efforts have been performed during the last two decades to accurately determine the Young’s modulus of collagen fibrils at the nanoscale, as it has been proven that mechanical alterations of collagen are related to various pathological conditions. Different contact mechanics models have been proposed for processing the force–indentation data based on assumptions regarding the shape of the indenter and collagen fibrils and on the elastic or elastic–plastic contact assumption. However, the results reported in the literature do not always agree; for example, the Young’s modulus values for dry collagen fibrils expand from 0.9 to 11.5 GPa. The most significant parameters for the broad range of values are related to the heterogeneous structure of the fibrils, the water content within the fibrils, the data processing errors, and the uncertainties in the calibration of the probe. An extensive discussion regarding the models arising from contact mechanics and the results provided in the literature is presented, while new approaches with respect to future research are proposed.

## 1. Introduction

**Figure 1:**
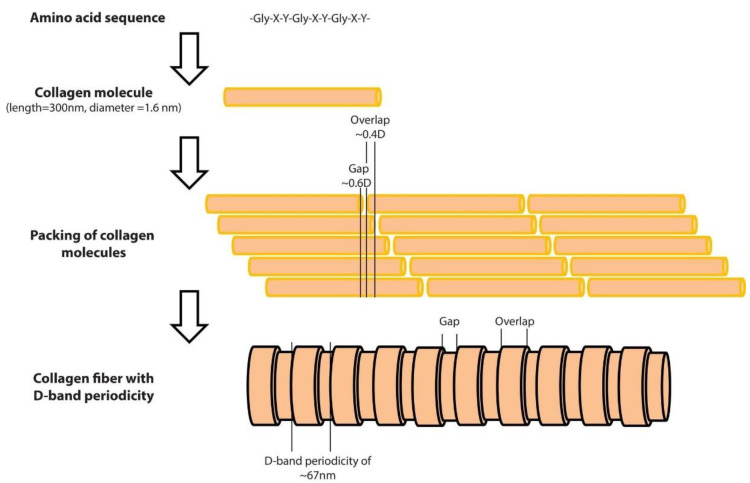
The structure of a collagen fiber type I, from the amino acid sequence to the D-band periodicity. As presented at the top of the figure, the collagen molecules consist of three amino acid chains. The length of the collagen molecule is 300 nm, while its diameter is 1.6 nm. The amino acid chains form rod-shaped triple helices which are assembled to form collagen molecules. Molecules are packed in a quarter-staggered fashion in order to form fibrils with a pattern that is known as the D-band periodicity. This pattern consists of overlapping and gap regions; it is a repeating banding pattern of about 67 nm as shown at the bottom of the figure.

Collagens are the major proteins in mammals (almost 30% of the total mammalian protein) [1]. The collagen superfamily consists of more than 50 collagens and collagen-like proteins, with the fibrous collagens, including collagen I, tending to be of most interest [2,3]. Collagen molecules are composed of three polypeptide chains that contain the repeating amino acid motif (Gly-X-Y), where X and Y can be any amino acid. The molecules of collagen type I, which is the major protein in the extracellular matrix [4] form rod-shaped triple helices assembled to form fibrils [4], which are then properly aligned in order to form bundles and fibers [4]. A unique and interesting characteristic of collagen fibers is the fact that collagen molecules are packed in a quarter-staggered fashion so as to form the D-band periodicity, which is a repeating banding pattern of about 67 nm, depending on the tissue (Figure 1) [1]. Collagen fibrils are the elementary building blocks in many tissues and organs, and play a crucial role in a number of pathological conditions [1]. Furthermore, collagen is considered one of the most widely used biomaterials, mainly due to its unique properties such as the ability of self-assembly, bio-compatibility, bio-degradability, and non-toxicity [5]. 

The mechanical properties of individual collagen fibrils have been extensively explored during the last decades using various methods such as Brillouin spectroscopy [6,7], force spectroscopy [8], X-ray data [9], microelectromechanical systems (MEMS) [10,11,12], steered molecular dynamics simulation of tropocollagen-like molecules [13], and Atomic Force Microscopy (AFM) nanoindentation [14,15,16]. The AFM nanoindentation became the leading technique regarding the characterization of biological samples at the nanoscale due to its great potential to be employed in real clinical activities. In particular, the nanomechanical characterization of individual collagen fibrils at the nanoscale has been related to new methods for the accurate and early diagnosis of cancer [17] and osteoarthritis [18]. Thus, significant scientific interest towards the accurate determination of collagen fibrils Young’s modulus using AFM nanoindentation has arisen during the last two decades. However, the reported values of Young’s modulus in the literature vary significantly. In particular, Grant et al. used Hertzian mechanics (sphere–sphere contact) to calculate the Young’s modulus of type I collagen fibrils obtained by bovine Achilles tendon [19]. The Young’s modulus resulted in 1.9 ± 0.5 GPa for dry fibrils and in 1.25 ± 0.1 MPa for fibrils in buffer solution [19]. Wenger et al. calculated the Young’s modulus of dry collagen type I fibrils from rat-tail tendon using the Oliver and Pharr analysis (sphere–elastic half space interaction) [20]. Their results were in the range of 5–11.5 GPa [20]. Heim et al. performed AFM nanoindentation experiments on collagen type I fibrils in air environment obtained from Cucumaria frondosa [21]. The Young’s modulus resulted in 1–2 GPa. The contact mechanics model in their analysis was the Hertz model (sphere–cylinder contact) [21]. Minary-Jolandan and Yu determined the Young’s modulus on overlapping (~2.2 GPa) and gap regions (~1.2 GPa) on dry collagen fibrils (type I) obtained by bovine Achilles tendon using Hertzian analysis (sphere–cylinder contact) [22]. Yadavally et al. used collagen type I from calf skin in their experiments. Using Hertzian analysis (sphere-sphere contact), they calculated the Young’s modulus equal to 1.9 ± 0.5 GPa for dry and 1.2 ± 0.1 MPa for hydrated collagen fibrils [23]. Andriotis et al. performed experiments on dry collagen type I fibrils from wild type mouse tail tendon (7.0 ± 1.5 GPa using Hertzian analysis, sphere-sphere contact and 9.4 ± 1.7 GPa using the Oliver and Pharr method), from rat tail tendon (3.2 ± 1.1 GPa, Oliver and Pharr analysis) and from human bronchial biopsies (6.6 ± 0.7 GPa, Oliver and Pharr analysis) [24]. Kontomaris et al. tested collagen type I fibrils from bovine Achilles tendon in air environment and the results were 0.9–1.5 GPa [25]. For their analysis the Oliver and Pharr model regarding the interaction of a sphere with an elastic half space was employed. Andriotis et al. used collagen from tail tendon of a two-month old mouse, and the results were 1–10 GPa [26] using the Oliver and Pharr method.

**Figure 2:**
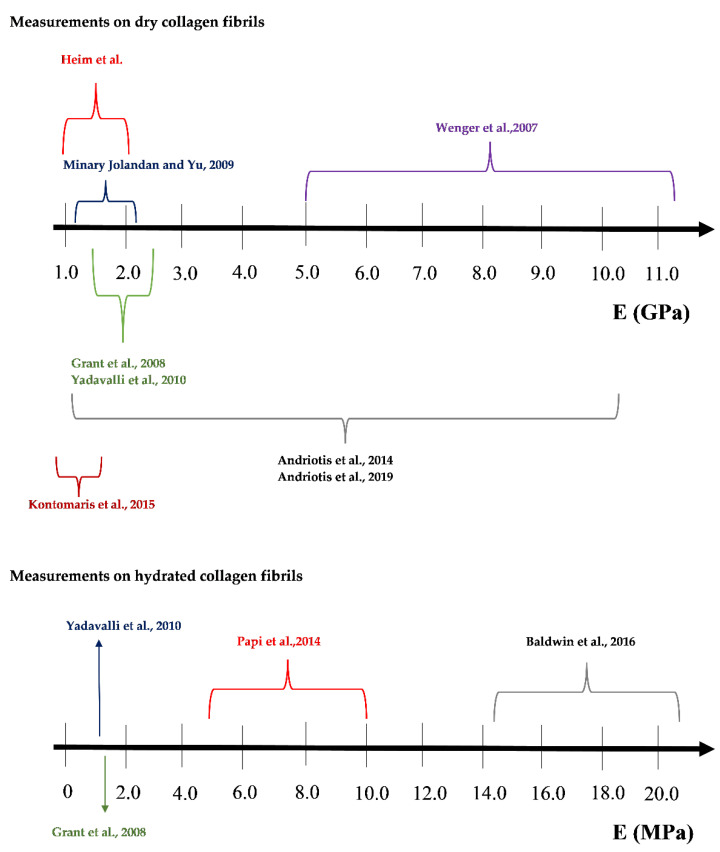
The range of Young’s modulus values in the literature [19,20,21,22,23,24,25,26,27,28].

Papi et al. used the PeakForce Quantitative Nanomechanical Property Mapping (PFQNM) to characterize the nanomechanical properties of collagen fibrils in sclera (type I collagen is the predominant form of collagen in sclera) [27]. The model that was used for processing the force curves was the Derjaguin–Muller–Toporov (DMT) model [27] and the results were in the range 5–10 MPa. Baldwin et al. presented Young’s modulus maps of normal and kinked hydrated collagen fibrils [28]. In case of normal fibrils the Young’s modulus resulted in 17.3 ± 3.9 MPa [28]. The analysis was performed using Sneddon’s model [28]. Interesting research has been also applied to collagen rich tissues. Kazaili et al. measured Young’s modulus of 5 μm thick corneal sections and the Young’s modulus resulted in the range 2 to 2.45 GPa [29]. For their analysis, the PFQNM mode was used in air environment [29]. The range of Young’s modulus values in the literature is also presented in Figure 2.

As already mentioned, the accurate nanomechanical characterization of individual collagen fibrils is crucial in order to use AFM as a diagnostic tool for various diseases [16,17,18]. Thus, in this paper, the reasons for such an extended range of Young’s modulus values in the literature are presented and discussed. The variability of the Young’s modulus values of single collagen fibrils is related to the dehydration state of the fibril [24], to errors in data processing [30], and to uncertainties regarding the AFM probe calibration procedures (the determination of the spring constant of the AFM cantilever and the exact shape and size of the tip) [20]. Apart from the water concentration within the fibril, it is significant to note that even for dry collagen fibrils the range of the resulting Young’s modulus values is extremely broad (even when using the exact same experimental conditions). In particular, the range of Young’s modulus values regarding individual dry collagen fibrils is 0.9–11.5 GPa [20,24,25,26]. This review focuses on the methods that are being used for the processing of the force–indentation data in indentation experiments in air environment, and analyzes the factors that lead to such an extended variation in the Young’s modulus values. It is known that environmental conditions affect the results of AFM data on biological samples (e.g., humidity affects the tip–surface interaction and also alters the sample’s mechanical properties) [31]. In addition, according to Quigley et al., a dehydration–rehydration cycle leads to a decrease in radial modulus [32]. Thus, in order to reduce the factors that affect the results, the discussion in this paper concerns only measurements performed in air. Finally, new research approaches are proposed towards an accurate gold standard nanomechanical characterization method in the future.

## 2. Data Processing

### 2.1. Collagen Sample Preparation

In the literature, a number of different experimental protocols can be found in order to prepare collagen-based specimens for nanoindentation experiments. However, they can be grouped into two major categories. The first method is the AFM experiments on collagen fibers derived from fresh tissue, the so-called native collagen. In this case, collagen fibrils are dissected from collagen rich tissues, such as rat tail tendons [20]. Tissue samples such as tendons are sectioned with scalpels and washed with deionized water or phosphate-buffered saline. Subsequently, bundles of collagen fibers are collected with tweezers and then deposited on clean substrates such as microscope glass slides. Depending on the study, the sample can be dried (e.g., air dry) or they can be immersed in PBS for performing the experiments dry or hydrated, respectively. The second method is the reconstruction of collagen fibers/fibrils from a collagen solution. Such collagen solutions can be found as commercially available solutions [33] or they can be prepared from other commercially available products such as collagen powders and lyophilized collagen [25,33,34,35,36,37,38]. There are a number of different collagen sources for these products, such as bovine Achilles tendon, calf skin, mouse/rat tail, etc. In these protocols, the first step is the preparation of a collagen solution with the final concentration. For the preparation of the solution, acetic acid is used for the lyophilized/powder collagens, while dilutions with PBS or other buffers is used for collagen solutions. In any case, the pH should be controlled (for example with NaOH). Subsequently, a number of different approaches can be used for the preparation of the samples, such as drop casting, the spin coating process, and the use of hydrodynamic flow [25,33,34,35,36,37,38]. As substrates, fresh cleaved mica discs are preferred, as they present a flat surface for AFM experiments. Again, the sample can be characterized either in air (dry) or liquid (e.g., in PBS) conditions.

### 2.2. The Calibration of Probe Parameters

For the accurate quantitative characterization of biological samples, the calibration of the probe parameters is a mandatory procedure. Firstly, a sensitivity calibration (nm cantilever deflection per Volt signal of the laser detection system) is important to be performed. In particular, a force vs. distance curve on a clean, hard surface (e.g., mica or glass) should be acquired [33]. The deflection V is measured directly by the system’s position-sensitive split photodiode detector [33]. Then, the deflection sensitivity is determined by this force vs. distance curve by simply positioning two cursors on its contact part [33]. With respect to the spring’s constant calibration, the most reliable method is the thermal noise method [33]. In addition, it is also important to measure the exact size of the indenter before a nanoindentation experiment. The aforementioned procedure can be performed using an AFM calibration grating [39] or SEM imaging [40]. An alternative to laser detection system is the tuning fork force sensor detection system [41,42,43]. In this case, the AFM tip is mounted perpendicular to a tuning fork so that the tip oscillates perpendicular to the sample’s surface [41]. The advantage of this system compared to optical ones is that the high spring constant of the tuning fork tine permits very small oscillation amplitudes (~0.1 nm) [41]. Thus, it can offer an improved spatial resolution.

### 2.3. Constructing a Force–Indentation Curve and Curve Fitting

When performing a nanoindentation experiment using AFM, the experimental data are presented in the form of a piezo-displacement–cantilever’s deflection curve [44]. Subsequently, the cantilever’s deflection signal in Volts is multiplied with the calibrated spring’s constant, and results in the applied force on the sample [44]. However, the indentation depth for each force value should be determined; in other words, the construction of a force–indentation curve is a mandatory procedure in order to find the Young’s modulus of the sample.

**Figure 3:**
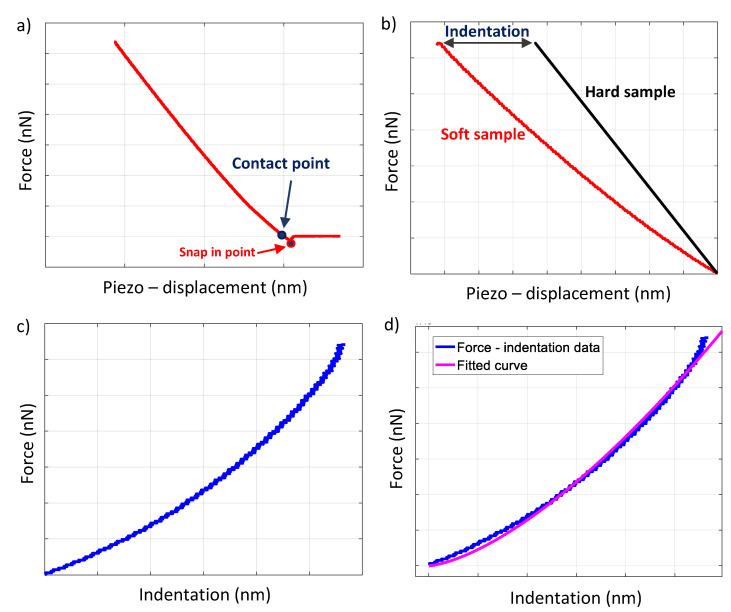
Data processing. (**a**) A force–piezo-displacement curve is obtained on the sample of interest and the contact point is determined. (**b**) The force-piezo-displacement curves for the sample of interest (soft sample) and for a reference material that is not being deformed by the tip (hard sample). (**c**) The piezo-displacements between the soft and the hard sample are subtracted and a force–indentation curve is created. (**d**) An appropriate model from contact mechanics is fitted to the experimental force–indentation curve.

The procedure is briefly presented in Figure 3. Firstly, a force vs. piezo-displacement curve on the sample of interest is acquired, and the contact point between the AFM tip and the sample is determined. In the case of force curves in hard samples, the contact point is usually easily determined as presented in Figure 3a. It should be noted that useful software platforms are available for the contact point determination (and for processing the raw force–indentation data), such as the AtomicJ software [45]. At this point, it is significant to distinguish the contact point from the snap-in point in Figure 3a. During sample’s approach towards the AFM tip, the long-range attractive force causes the tip to snap in toward the sample surface (Figure 3a). The snap in point represents the initial point of contact (assuming that the tip lies perfectly on the surface after snapping in without any deformation of the sample surface) [20]. However, in most of the cases of AFM indentation experiments on collagen, the contact point is considered as the point where the adhesion balances the repulsive force (zero cantilever deflection). The reason is that, after the aforementioned point, the indentation of the tip into the sample begins.

After the determination of the contact point, the difference between the piezo-displacement for the sample of interest and for a hard reference material is calculated for each force value. The aforementioned difference is the indentation depth on the sample [44] (Figure 3b,c). The last step is to fit the force–indentation data to an appropriate contact mechanics model depending on the shape of the indenter and the shape of the sample (Figure 3d) [44,46]. The curve fitting can be performed with various software (e.g., the AtomicJ software [45]). For example, in the case of an indenter that can be considered as a paraboloid of revolution, or for a spherical indenter (for small indentation depths compared to the tip radius), the loading force–indentation data should be fitted to the following equation (assuming a purely elastic contact) [47,48]:

(1) $$F=\frac{4}{3}\frac{E}{\left(1-v^{2}\right)}R^{1/2}h^{3/2}$$

In Equation (1), *F* is the applied force on the sample, *h* is the indentation depth, *R* is the tip radius, and *E* and *ν* are the Young’s modulus and the Poisson’s ratio, respectively. Thus, the Young’s modulus can be determined as a fitting parameter, assuming the sample’s Poisson’s ratio is known. It must also be noted that Equation (1) is valid when the sample can be approximately considered as an elastic half space and the AFM tip is orders of magnitude harder compared to the sample (which is usually the case when testing biological samples with AFM).

As already mentioned, Equation (1) is valid for spherical indentations if *h* ≪ *R* (as it was derived for the interaction between a paraboloid of revolution and an elastic half space) [49,50,51]. The accurate solution regarding the interaction of a rigid sphere and an elastic half space was provided by Sneddon [52,53]:

(2) $$F=\frac{E}{2\left(1-v^{2}\right)}\left[\left(r_{c}^{2}+R^{2}\right)ln\left(\frac{R+r_{c}}{R-r_{c}}\right)-2r_{c}R\right]$$

In Equation (2), $r_{c}$ is the radius at contact depth ($h_{c}$) (i.e., the depth at which contact is made between the half space and the sphere) [20]. In addition, the indentation depth is related to the contact radius by the following equation [52]:

(3) $$ln\left(\frac{R+r_{c}}{R-r_{c}}\right)=\frac{2h}{r_{c}}$$

However, Equations (2) and (3) do not provide a direct relation between the applied force and the indentation depth. Thus, recently, a new equation was derived [54]:

(4) $$F=\frac{4ER^{1/2}}{3\left(1-v^{2}\right)}h^{3/2}A$$

In Equation (4),

(5) $$A=c_{1}+\overset{N}{\underset{M=2}{\sum }}\frac{3}{2Μ}c_{M}R^{\left(\frac{3}{2}-M\right)}h^{M-3/2}$$

In Equation (5), $c_{1},\;c_{2}$,…,$\;c_{N}$ are constant parameters [54]. For example, if *h/R* = 0.6, $c_{1}$ = 1.01, $c_{2}$ = −0.075, and $c_{3}$ = −0.1142. Extended information for the constants $c_{1}$,$\;c_{2}$,…,$\;c_{3}$ for any indentation depth is provided in [54].

As the indentation depth on collagen fibrils should be small to avoid substrate effects [24], the aforementioned equations can be also used for pyramidal indenters with a round tip apex. For example, when using a pyramidal tip with a tip radius equal to *R* = 20 nm, the data processing can be performed using Equation (1) if the maximum indentation depth is up to *R*/10 = 2 nm or with Equations (2)–(5) for indentation depths up to ~20 nm.

### 2.4. The Purely Elastic Sphere–Cylinder Interaction

**Figure 4:**
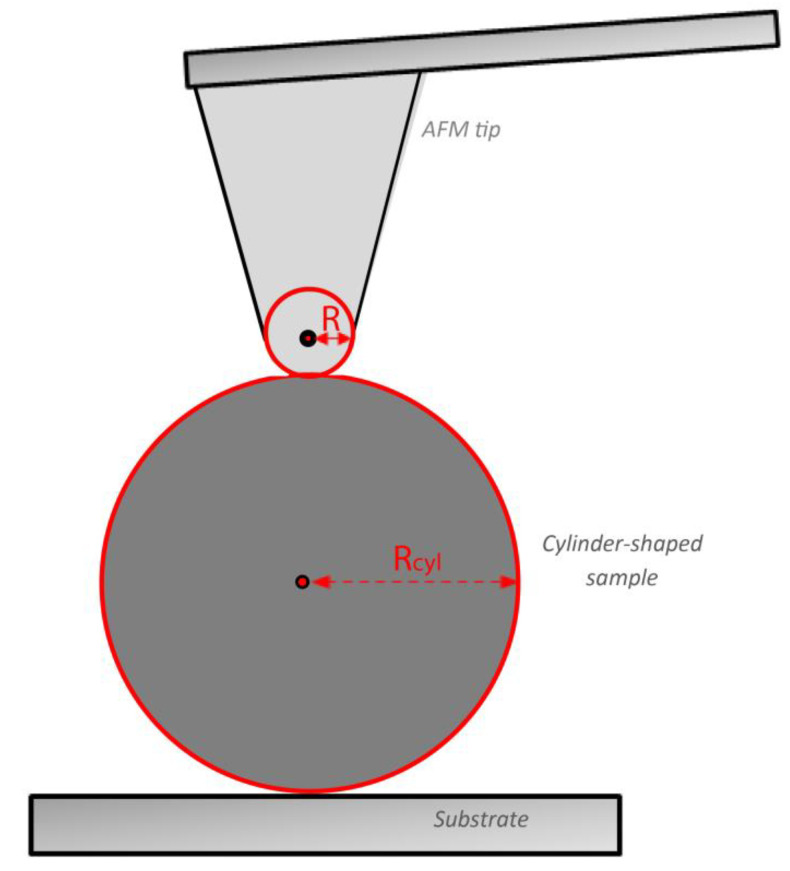
Indentation experiment on a cylinder-shaped homogeneous and isotropic sample. The AFM tip can be approximately considered as a rigid spherical indenter for small indentation depths.

The equations presented in Section 2.2 presuppose that the sample can be considered as an elastic half space [16,54,55]. In other words, the sample is significantly bigger than the AFM tip and presents a linear elastic behavior. Thus, they can be used only if the radius of the collagen fibril is extremely big compared to the tip radius [56,57]. A rational approximation to apply the equations of Section 2.2 is the fibril radius to be at least 5 times bigger compared to the tip radius [56,57]. However, from a rigorous mathematical perspective, when indenting a collagen fibril using an AFM tip the interaction can be modelled as the interaction between a rigid sphere, and a cylinder-shaped sample under the restriction that the tip apex can be considered as spherical (Figure 4). As previously reported [58], the projected area at contact depth is elliptic and the Young’s modulus can be calculated using the following equation:

(6) $$E=\frac{3}{4}\left(1-v^{2}\right)FR^{-1/2}h^{-3/2}Z$$

In other words, the data should be fitted to equation:

(7) $$F=\frac{4}{3}\frac{E}{\left(1-v^{2}\right)}R^{1/2}h^{3/2}\frac{1}{Z}$$

The term *Z* equals to [58]:

(8) $$Z=\frac{2k}{\sqrt{2}\pi }{\left(\frac{K^{3}\left(k\right)}{K\left(k\right)-E\left(k\right)}\right)}^{1/2}$$

where the term *k* is equal to $k=\sqrt{1-{\left(\frac{a}{b}\right)}^{2}}$, where *a* and *b* are the semi-axes of the contact ellipse between the bodies, the term K(k) is the complete elliptic integral of the first kind and is equal to $K\left(k\right)=\int_{0}^{1}\frac{dx}{\sqrt{\left(1-x^{2}\right)\left(1-k^{2}x^{2}\right)}}$, and E(k) is the complete elliptic integral of the second kind and is equal to $E\left(k\right)=\int_{0}^{1}\sqrt{\frac{1-k^{2}x^{2}}{1-x^{2}}}dx$ [57,58].

As can be easily seen from Equation (7), the approximation of a cylinder-shaped sample as a half space is validated in the limit Z→1 (in this case, $\frac{R}{R_{cyl.}}\to 0$). Furthermore, the $\frac{R}{R_{cyl.}}$ ratio is presented as follows [57,58]:

(9) $$\frac{R}{R_{cyl.}}=\frac{E\left(k\right)-\left(1-k^{2}\right)K\left(k\right)}{\left(1-k^{2}\right)\left[K\left(k\right)-E\left(k\right)\right]}-1$$

In addition, previous research showed that the term Z in Equations (6)–(8) can be expressed with respect to the ratio $\frac{R}{R_{cyl.}}$ using the following equation [57]:

(10) $$Z=c{{}^{\prime }}_{2}{\left(\frac{R}{R_{cyl.}}\right)}^{2}+c{{}^{\prime }}_{1}\left(\frac{R}{R_{cyl.}}\right)+c{{}^{\prime }}_{0}$$

In Equation (10), $c{{}^{\prime }}_{0}=1.002$, $c{{}^{\prime }}_{1}=0.223$, and $c{{}^{\prime }}_{2}=-0.046$ [57]. Thus, if the $\frac{R}{R_{cyl.}}$ ratio is known, the *Z*-term can be easily calculated using Equation (10) and the Young’s modulus of the cylinder-shaped sample can be determined as a fitting parameter using Equation (7). In Table 1, characteristic values of the *Z*-factor given by Equation (10) are presented for various values of the ratio $\frac{R}{R_{cyl.}}$. Similar approaches regarding experiments on collagen fibrils can be also found in the literature. Grant et al. [19] used the two spheres in contact approximation as follows:

(11) $$F=\frac{4}{3}\frac{E}{1-v^{2}}R_{eff\left(1\right)}^{1/2}h^{3/2}$$

In Equation (11), $R_{eff\left(1\right)}$ is the effective radius that equals to:

(12) $$R_{eff\left(1\right)}=\frac{RR_{cyl.}}{R+R_{cyl.}}=R\left(\frac{1}{\frac{R}{R_{cyl.}}+1}\right)=\frac{R}{X}$$

By substituting Equation (12) to Equation (11), it can be easily concluded that:

(13) $$F=\frac{4}{3}\frac{E}{1-v^{2}}R^{1/2}h^{3/2}\frac{1}{X^{1/2}}$$

In addition, Heim et al. [21] used as an effective radius:

(14) $$R_{eff\left(2\right)}=\sqrt{\frac{R^{2}R_{cyl.}}{R+R_{cyl.}}}=R\sqrt{\frac{1}{\frac{R}{R_{cyl.}}+1}}=\frac{R}{\sqrt{X}}$$

In this case,

(15) $$F=\frac{4}{3}\frac{E}{1-v^{2}}R_{eff\left(2\right)}^{\frac{1}{2}}h^{\frac{3}{2}}=\frac{4}{3}\frac{E}{1-v^{2}}R^{1/2}h^{3/2}\frac{1}{X^{1/4}}$$

**Figure 5:**
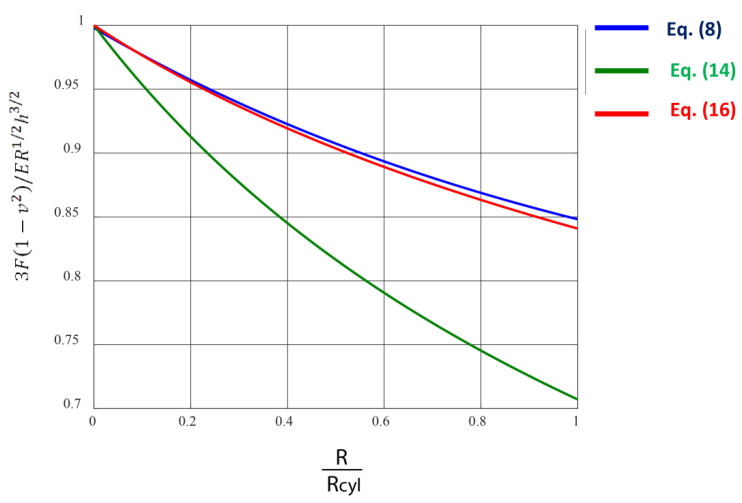
Plots representing different models for data processing. The $\frac{F}{\left(\frac{4}{3}\frac{E}{1-v^{2}}R^{\frac{1}{2}}h^{\frac{3}{2}}\right)}=f\left(\frac{R}{R_{cyl.}}\right)$ graphs using different models. The accurate model regarding the sphere–cylinder interaction is provided by Equation (7).

In Figure 5, the $\frac{F}{\left(\frac{4}{3}\frac{E}{1-v^{2}}R^{\frac{1}{2}}h^{\frac{3}{2}}\right)}=f\left(\frac{R}{R_{cyl.}}\right)$ graphs using Equations (7), (13) and (15) are presented. The model, which is closer to the accurate solution regarding the interaction between a rigid spherical indenter and a cylinder-shaped sample (i.e., Equation (7)), is the one presented by Equation (15), as can be clearly seen in Figure 5.

### 2.5. The Elastic–Plastic Contact

Pharr et al. [59] derived an equation that can be used to determine the Young’s modulus for any sample of interest when using an axisymmetric indenter on an elastic half space. In this case, the Young’s modulus is provided as follows:

(16) $$E=\frac{\sqrt{\pi }}{2}\left(1-v^{2}\right)\frac{S}{\sqrt{A_{c}}}$$

In Equation (16), *S* = *dF/dh* is the contact stiffness at the maximum indentation depth and *A_c_* is the projected area at contact depth between the indenter and the sample. It is significant to note that Equation (16) was initially derived for purely elastic contact [56,59,60]. For example, in the case of a parabolic indenter *A_c_* = *π*$r_{c}^{2}$ = *πRh* [30]. Thus, Equation (16) is written as follows:

(17) $$\frac{dF}{dh}=\frac{2E}{\left(1-v^{2}\right)}R^{1/2}h^{1/2}\;$$

The solution of the differential Equation (17) is the classic Hertz equation for parabolic indenters (absolutely identical to Equation (1)).

**Figure 6:**
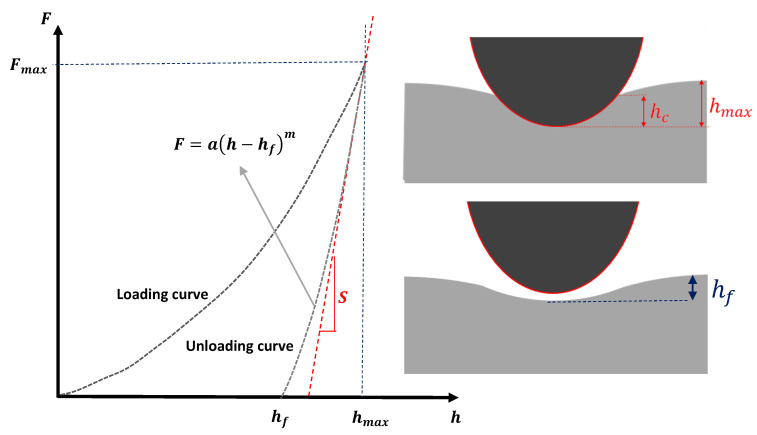
The elastic–plastic contact. The unloading force–indentation data can be fitted to Equation (18). The final depth (depth at which the deformation is permanent) is calculated as a fitting parameter. A simple method for fitting the unloading data can be found in [62]. The slope of fitted curve at the maximum indentation depth equals to the contact stiffness *S* (Equation (19)).

Thus, when testing a linear elastic sample using a parabolic indenter and there is no permanent deformation on the sample, Equations (1) and (16) are identical. However, Oliver and Pharr experimentally proved that Equation (16) can also extend its applicability in an elastic–plastic contact using only the unloading data [20,30,51,61]. In particular, it is assumed that only the elastic displacements are recovered during unloading [20,30,51,61]. The Oliver and Pharr method has been extensively applied in the determination of the mechanical properties of collagen fibrils. The first step is to fit the unloading data to an equation of the following form [20,51,61] (Figure 6):

(18) $$F=a{\left(h-h_{f}\right)}^{m}$$

where α, *h_f_*, and *m* are determined as fitting parameters. It must be also noted that *h_f_* represents the final indentation depth (i.e., the depth at which a permanent deformation occurs after the indenter is withdrawn). According to the initial analysis of Oliver and Pharr, the exponent *m* for most of the materials should be in the range 1.2 ≤ *m* ≤ 1.6 [51,61]. However, Wenger et al. showed that the exponent m can also take lower values as well for the case of collagen fibrils (e.g., *m* ≈ 1.1) [20]. A simple way to fit the unloading force–indentation data was recently presented [62]. Subsequently, the contact stiffness as defined in Equation (16) can be derived as the first derivative of Equation (18) with respect to depth (*h*) at the maximum indentation depth (*h_max_*):

(19) $$S={\frac{dF}{dh}|}_{h_{max}}=am{\left(h_{max}-h_{f}\right)}^{m-1}$$

The next step is to calculate the projected area at contact depth. Most of the indenters used in the characterization of biological samples are pyramidal with a round tip apex [44,46]. Usually, when indenting a collagen fibril, the maximum indentation depth should be a few nanometers to avoid the already mentioned substrate effect [24,63]. In this case, it can be assumed that the contact area is circular and is provided through the following equation [20]:

(20) $$A_{c}=\pi \left(2Rh_{c}-h_{c}^{2}\right)\;$$

In Equation (20), *h_c_* represents the contact depth (i.e., the depth at which contact is made between the indenter and the sample). The contact depth can be determined as follows [20,51]:

(21) $$h_{c}=h_{max}-\varepsilon \frac{F_{max}}{S}\;$$

The factor *ε* is determined using the equation below [20,51,62]:

(22) $$\varepsilon =m\left\{1-\frac{2\Gamma \left[\frac{m}{2\left(m-1\right)}\right]}{\sqrt{\pi }\Gamma \left[\frac{1}{2\left(m-1\right)}\right]}\left(m-1\right)\right\}\;$$

In Equation (22), *Γ* is the gamma function. The parameter *ε* depends on the indenter’s geometry. For example, in case of a perfect parabolic indenter, *m* = 1.5 and Equation (22) results in *ε* = 0.75. Another approach for the determination of contact area was provided by Andriotis et al. [24] who used a Berkovich tip (which is a three-sided pyramid). In particular, the tip was mounted on a special sample holder exposing the tip apex towards the AFM cantilever, and the tip apex was then imaged using AFM tapping mode [24]. Finally, numerical integration of the *Z*-sensor channel data delivered the area function of the indenter. The contact area was then expressed with the polynomial [24]:

(23) $$A_{c}=kh^{2}+\lambda h\;$$

where *k* and *λ* are fitting parameters.

It is also significant to note that the previously presented analysis regarding the determination of the contact area assumed that the fibril’s diameter is much bigger compared to the dimensions of the tip (elastic half space approximation). In cases in which the diameter of the fibril is comparable to the tip radius, the best approach is to consider an elliptical contact area [64] as follows:

(24) $$A_{c}=\frac{\pi Rh}{Z^{2}}$$

where *Ζ* is given by Equation (10). Using Equation (24), Equation (16) can be written as follows [64]:

(25) $$E=\frac{\sqrt{\pi }}{2}\left(1-v^{2}\right)\frac{S}{\sqrt{\pi Rh}}Z$$

### 2.6. Mechanical Properties Maps

The models as described in previous sections can be used for multiple measurements over a selected region of a collagen fibril. Using this approach, a Young’s modulus map can be created. For example, in [20] a Young’s modulus map, created by multiple measurements using the Oliver and Pharr approach, is shown. As an alternative, a Young’s modulus map can be created using the force scanning method [65]. In this case, a selected area is scanned (using AFM contact mode) N times using different set point forces. Thus, N images are created; each is characterized by z-values (which represent the height of each point). By subtracting these values by an arbitrary contact point height, the indentation depths are obtained and arrays that consist of indentation values at every point are created. Thus, at each point, the force–indentation curve is available. The data can then be processed using the Hertz model [65]. A Young’s modulus map using the force scanning method on a collagen fibril type I can be found in [66]. In addition, the PeakForce QNM method, which is based on the Peak Force Tapping mode, can be used instead [27,29]. In this case, the probe is periodically in contact with the surface of the sample in order to apply a periodic force; thus, a force–separation curve is created for each ‘tap’ of the tip on the surface [27,29]. The method is based on controlling the maximum force (peak force) on the sample at each point. It is significant to note that the basic principles in this case are comparable to the principles behind the classic force displacement curves of the classic nanoindentation procedure. However, in this case, the DMT model is used, which is an extension of the Hertz model (the difference is that the DMT model takes also into consideration the adhesive forces between the tip and the sample) [67]. The magnitude of the adhesive force is often estimated using the Derjaguin approximation [67]:

(26) $$F_{ADH.,DMT}=2\pi R_{eff.}W_{ADH.}\;$$

Equation (26) relates the energy between two parallel surfaces to the force in a sphere-on-flat or sphere-on-sphere geometry [67]. Thus, the tip-sample force is provided as follows [44]:

(27) $$F_{tip-sample}=\frac{4E}{3\left(1-v^{2}\right)}R_{eff.}^{1/2}h^{3/2}+F_{ADH.,DMT}$$

## 3. A Discussion Regarding the Extended Range of Young’s Modulus Values Found in the Literature

In Section 2, the approaches for testing the mechanical properties of individual collagen fibrils using the AFM indentation method were clearly presented and discussed. In this section, the reasons for such an extended range of Young’s modulus values in the literature, even when the exact same protocol is used, will be discussed.

### 3.1. The Mechanical Heterogeneity of Collagen Fibrils

Firstly, it must be clarified that collagen fibrils are not linear elastic solids. In fact, the diameter of each fibril changes along the axial direction (the collagen fibril consists of an alternating gap and overlapping regions), with a highly reproducible D-band periodicity of approximately 67 nm [22,25]. The aforementioned heterogeneity results also in a mechanical heterogeneity. In particular, Minary-Jolandan and Yu performed experiments on type I collagen fibrils prepared from bovine Achilles tendon and calculated the Young’s modulus on overlapping regions ~2.2 GPa, while on gap regions ~1.2 GPa [22]. The analysis was performed using the theory regarding the sphere–cylinder purely elastic interaction [22]. Kontomaris et al. reported similar results regarding the aforementioned mechanical heterogeneity. In their experiments (which were also conducted on type I collagen from bovine Achilles tendon) they found a Young’s modulus of ~1.1 GPa for overlapping regions and ~0.9 Gpa for gap regions [25]. In their analysis, the Oliver–Pharr method for elastic–plastic contact using a spherical indenter was used [25]. It is also significant to note that mechanical properties maps of collagen fibrils in terms of Young’s modulus have been previously presented [66]. The mechanical heterogeneity of collagen due to overlapping and gap regions is a significant factor related to the broad distribution of Young’s modulus values in the literature and is highly related to the dehydration state. In particular, Spitzner et al. investigated how the water influences the mechanical properties of individual type I collagen fibrils on the nanometer scale. In particular, the mechanical contrast between overalapping and gap regions is small in dry state, while in hydrated state the differences are significant [68].

### 3.2. Errors in Calibration Procedures

The first paper with a significant effort to provide an answer regarding the extended range of Young’s modulus values, even when testing the same collagen fibril, was presented by Wenger et al. [20]. In their research, it was found that the Young’s modulus of individual dry collagen fibrils resulted in the range 5 GPa–11.5 GPa. The model was used for data processing was the Oliver–Pharr one for an elastic–plastic contact using spherical indenters (pyramidal indenters with round tip apex). The error in the spring’s constant determination was estimated to be of ~5%. In addition, according to Wenger et al. [20] assuming an uncertainty of ~20% regarding the determination of the tip radius and an uncertainty of ~10% regarding the indentation depth the error should not exceed ~30%. However, at this point, it is significant to note that the error due to the contact area determination is probably significantly smaller compared to what is mentioned above. According to Oliver–Pharr model, the Young’s modulus is calculated using Equation (16). Assuming that the real contact area is $A_{c}$ and the calculated contact area is $A_{c}^{’}=1.3A_{c}$ (i.e., 30% error), the Young’s modulus results in:

$$E^{{}^{\prime }}=\frac{\sqrt{\pi }}{2}\left(1-v^{2}\right)\frac{S}{\sqrt{1.3A_{c}}}\approx 0.88E$$

where E is the real Young’s modulus. Thus, due to a ~30% uncertainty in the value of contact area, the uncertainty in Young’s modulus is only ~12%. Moreover, Wenger et al. concluded that the error related to the nanoindentation depth cannot be avoided, as in collagen fibrils experiments there are small indentation depths of only few nanometers [20,24] that are very sensitive to the absolute error in the measurement of the cantilever deflection of ~0.5 nm [20]. In addition, they reported that the combination of the contact area and the fibril stiffness uncertainties resulted in a random error of ~20% for the reduced modulus (i.e., for the parameter $E/\left(1-v^{2}\right)$*)* [20]. In addition, they assumed a circular contact area between the AFM tip and the fibril (as the measured fibrils had a radius at least five times the size of the tip radius), not an elliptical one. However, the estimated error due to the shape of the contact area was no bigger than 3% [20]. In addition, the attractive interfacial forces (long-range attractive force, adhesion force) between the AFM tip and the sample surface may result in errors regarding the Young’s modulus calculation. However, inaccuracies related to the attractive interfacial forces are usually not taken into account, as their contribution is small (adhesion was ~<5% of the maximum load) compared to other systematic errors [20]. The conclusion of the aforementioned very interesting analysis is that the errors in calibration process cannot solely explain the extended range in Young’s modulus values.

### 3.3. Errors in Fitting Process

**Figure 7:**
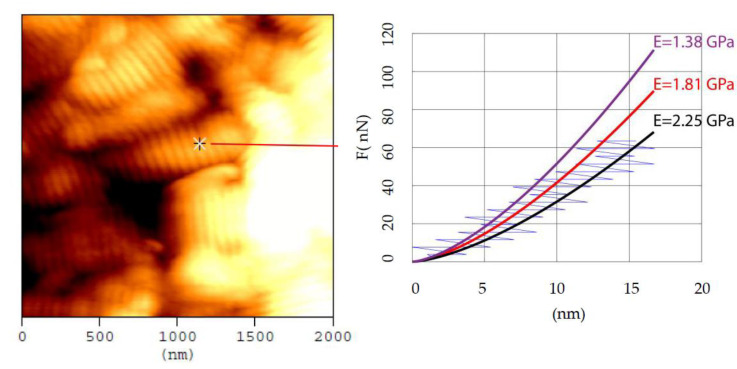
Obtaining a force–indentation curve on a collagen fibril. The protocol used for preparing the collagen type I, from bovine Achilles tendon, fibrils presented in the topography image is described in [25]. AFM experiments were conducted using a commercial microscope (CP II, Veeco Bruker, Santa Barbara, CA) in contact mode. For both imaging and indentation tests, pyramidal tips (MLCT tips constructed by Bruker) were employed. These tips have a nominal tip radius of approximately 20 nm on V-shaped cantilevers and a spring constant of 0.6 N/m. Three force–indentation curves on a collagen fibril with radius of ~200 nm were plotted. Depending on the range of data that will be used for processing, the Young’s modulus values vary significantly. The reason is that a collagen fibril is not a linear, elastic solid.

Another significant aspect of the extended range of Young’s modulus values is related to the fitting process regarding the force–indentation data. As previously mentioned, when a purely elastic contact between the AFM tip and the sample is being assumed, then the loading force–indentation data should be fitted to the appropriate model. For example, Equation (1) should be used assuming the interaction between a spherical indenter and a fibril with a significantly bigger radius compared to the tip radius for small indentation depths; Equation (4) should be used for bigger indentation depths; and Equation (7) should be used for thin fibrils. The loading force–indentation data, in the case of indenting a fibril using a pyramidal indenter with round tip apex, are shown in Figure 7 (see also a typical force–indentation depth on a collagen fibril in [23]). It is widely acceptable that the indentation depth should be only a few nanometers [20,23,24]. However, the exact maximum indentation depth significantly affects the fitting procedure. To better explain the abovementioned statement, the loading force–indentation data on a dry collagen fibril are displayed in Figure 7.

In Figure 7, three different fitted curves are shown for the loading force–indentation data obtained at the point indicated in the topography image. Assuming that only the first 5 nm are taken into account (purple curve), the calculated Young’s modulus resulted in 1.38 GPa. Assuming the first 9 nm, the appropriate fitting is the red curve, thus the Young’s modulus results in 1.81 GPa. For indentation depths comparable to the tip radius, the most appropriate fitting curve is the black one; in this case, the calculated Youngs modulus results in 2.25 GPa. The aforementioned calculations were performed assuming Poisson’s ratio v=0.5 [24] and purely elastic contact between a sphere and a half space (the radius of the fibril was ~200 nm which is 10 times bigger than the tip radius, thus the elastic half space approximation is rational) [20,56,57]. In other words, Equation (4) was used. It is interesting that the third calculation (i.e., 2.25 GPa) is ~63% bigger compared to the first one (1.38 GPa). Thus, it is obvious that there are significant uncertainties when fitting the force–indentation data that can justify up to a point the significant range of values when using the same experimental protocols (even when testing the same fibril). An interesting analysis which shows the extended number of different curves that can be used to describe the same collagen fibril is also shown in [22]. From the aforementioned analysis, it is clearly shown that the Young’s modulus on a specific point will differ based on the maximum indentation depth that will be used for data processing [22].

### 3.4. Errors due to Various Misuses of Contact Mechanics Models

Equation (25) proves that the error regarding the Young’s modulus calculation when considering a ‘thin’ fibril as a half space is not negligible. For example, in the case of the fibril’s radius equaling the tip radius, Z = 1.178 (Table 1). Thus, if the parameter Z is ignored, then the calculated Young’s modulus will be E/Z = 0.8489 E which is 15% smaller compared to the real value. In addition, an interesting analysis presented by Andriotis et al. [24] showed that the Hertz model results are 34% smaller compared to the Oliver–Pharr results for the same fibril [24]. The explanation of this phenomenon may have two different aspects [30]. The first is based on assumptions regarding the contact area. For example, if Equation (1) is used, it is assumed that the contact area between the spherical indenter and the sample equals to $A_{c}=2\pi Rh_{c}$ (where $h_{c}=h/2$) [30]. On the contrary, when using Oliver–Pharr analysis, the real contact area taken into account is $A_{c}=2\pi Rh_{c}-\pi h_{c}^{2}$. Thus, as previously mentioned, a 30% error in contact area leads to 12% error in Young’s modulus value. According to Equation (16), $E\sim 1/\sqrt{A_{c}}$, the Young’s modulus when using the Oliver–Pharr method will be, as a result, ~12% bigger. In order to minimize this difference, Equation (4) must be used instead of Equation (1). In addition, the possibility of small viscoelastic effects that were ignored could result in ~25% additional error [30].

### 3.5. The Importance of Research on Collagen

The significant efforts for the accurate nanomechanical characterization of individual collagen fibrils are related to significant medical applications such as cancer and osteoarthritis diagnosis [17,18]. In addition, collagen, and particularly collagen mutations, are related with a number of diseases such as osteogenesis imperfecta, chondrodysplasias, osteoporosis, and a number of syndromes (e.g., Ehlers–Danlos, Alport, and Knobloch). Furthermore, structural variations of collagen at the nanoscale are also related with various pathological issues [69,70], while collagen alterations in terms of structure, orientation, and mechanical properties have been found to play a crucial role in desmoplastic solid tumors, such as breast and pancreatic cancers [71,72,73,74,75]. In addition, AFM seems to be the most appropriate tool for the investigation of the influence of radiations, e.g., UV irradiation, radiofrequency radiation, etc. (either from nature or from medical activities), on tissues which contain collagen [38,76,77,78,79,80,81,82]. It is widely accepted that the UV irradiation from the sun affects human health; the chronic exposure to UV radiation can be harmful and probably lead to sunburn, photoaging, corneal damage, and carcinogenesis [83,84]. In addition, UV irradiation is also used for science purposes (e.g., cross-linking and sterilizing procedures) [85,86,87]. As UV can cause significant structural and mechanical alterations in collagen properties [88,89,90,91], it is significant to investigate these alterations using cutting edge scientific methods such as the AFM indentation method. Furthermore, the nanomechanical properties determination of the basilar membrane after cochlear implantation reveals localized stiffening. Thus, significant applications regarding tissues that contain collagen enhance the need for investigating collagen fibrils’ properties [92].

From the abovementioned paradigms, it is clear that the accurate nanocharacterization of the mechanical properties of individual collagen fibrils is crucial in order to apply AFM techniques in diagnostics and in real clinical activities.

## 4. Conclusions

This review paper focuses on the analysis of the methods that have been previously used for data processing of the force indentation data on individual collagen fibrils. In addition, an extensive discussion regarding the reasons that lead to such an extensive range of Young’s modulus values in the literature (even when the same fibril, the same protocol, and the same contact mechanics model are used) was presented. The main factors that lead to significant uncertainties and errors can be separated into 4 main categories:

- Errors in calibration procedures.
- The fitting processes.
- The contact mechanics models.
- The structural and mechanical heterogeneity of collagen fibrils.

It is significant to always keep in mind that collagen fibrils are not linear elastic solids; thus, apart from errors in experimental procedures or in data processing, the extended Young’s modulus values are an outcome of the heterogeneity, anisotropy, and water content of fibrils (as the other uncertainties can be controlled up to some extent). Minimizing the errors in data processing is extremely important in AFM procedures; however, especially for the case of heterogeneous and anisotropic samples, the need for new mathematical approaches for data processing is crucial for a reliable mechanical nano-characterization of materials that will enable the use of AFM methods in real clinical activities. Thus, in order to use the AFM nanoindentation method in real clinical practice in the future, it is significant to create new mathematical models targeted on describing the heterogeneous behavior of the collagen fibrils and to perform different measurements in order to obtain functions that relate the mechanical properties of individual fibrils to the indentation depth, to the water content, and to the alternating overlapping and gap sequence. In other words, it cannot be expected to describe highly mechanical heterogeneous samples with a single Young’s modulus value with high accuracy (for example, even if the tip radius, the spring’s constant, and the maximum indentation depth are determined with 99.9% accuracy, the extended range of Young’s modulus values in the literature will remain); functions and mechanical patterns should be determined for every biological sample (an example regarding functions that can describe the heterogenous mechanical behavior of cells can be found in [93]). The average Young’s modulus is probably the most appropriate physical quantity to describe the mechanical heterogeneity of collagen fibrils. It is certain that there is a specific mechanical pattern that can accurately describe a collagen fibril. In particular, it has been already proved that the overlapping regions are ‘stiffer’ compared to the gap regions [22,25] and the measured mechanical properties are not being affected by the fibrils diameter [20,24]. Thus, future research should reveal how the average Young’s modulus [93] changes with respect to:

- the indentation depth (i.e., across the radial direction of fibrils);
- the direction along the fibril (it is expected to lead in a periodic function due to the alternating gap and overlapping regions);
- the moisture content within the fibril.

The abovementioned experiments will probably lead to an accurate characterization of the mechanical properties of collagen fibrils and to the application of AFM techniques in real clinical activities and diagnostics related to changes in collagen fibrils’ properties.

## Figures and Tables

**Table 1:** Correction factor Z for the interaction between a sphere and a cylinder.

$R_{cyl.}$	Z–Factor (Equation (10))	$R_{cyl.}$	Z–Factor (Equation (10))
0.100	1.024	0.850	1.158
0.150	1.034	0.900	1.165
0.200	1.045	0.950	1.172
0.250	1.055	1.000	1.179
0.300	1.065	1.050	1.185
0.350	1.074	1.100	1.191
0.400	1.084	1.150	1.197
0.450	1.093	1.200	1.203
0.500	1.103	1.250	1.208
0.550	1.111	1.300	1.214
0.600	1.112	1.350	1.219
0.650	1.128	1.400	1.223
0.700	1.135	1.450	1.228
0.750	1.143	1.500	1.232
0.800	1.151		

## Data Availability

Not applicable.
